# Evaluation of the implementation of infection control policies in health facilities in the Popokabaka health district in the Democratic Republic of Congo

**DOI:** 10.3205/dgkh000542

**Published:** 2025-04-30

**Authors:** Fraste Kaswij Muswiya, Martin Mutuza Bakuzeza, Dalau Nkamba Mukadi

**Affiliations:** 1 Department of Environmental Health, Kinshasa School of Public Health, University of Kinshasa, Kinshasa, DR Congo; 2 Department of Community Health, Kinshasa School of Public Health, University of Kinshasa, Kinshasa, DR Congo

**Keywords:** preventive health, infection control, healthcare facilities, Popokabaka, Democratic Republic of Congo

## Abstract

**Background:**

Healthcare quality in health facilities relies on the implementation of and providers’ adherence to an effective infection control program. The aim of this study was to assess the implementation level of infection prevention and control (IPC) guidelines in healthcare facilities in a low-income country.

**Methods:**

This was a cross-sectional study conducted in 18 healthcare facilities of the Popokabaka health district in the Democratic Republic of Congo. Data were collected and analyzed following the IPC assessment framework developed by the World Health Organization (WHO). The framework consisted of eight different sections, of which each is scored up to 100. The level of implementation in each facility was assessed based on a scoring system: inadequate (0–200), basic (201–400), intermediate (401–600), and advanced (601–800).

**Results:**

The median score of all facilities was 181.3, IQR 145.0–228.1, with a range from inadequate to basic. Ten (55.6%) healthcare facilities had an inadequate IPC implementation level, while eight (44.4%) had a basic level. IPC education and training were the components that were the most poorly implemented in the surveyed facilities. None of these facilities had multimodal strategies to implement IPC interventions.

**Conclusion:**

The level of IPC guideline implementation in healthcare facilities of the Popokabaka health district remains basic as a result of low resource investment in the IPC program. It negatively impacts the quality of care and exposes patients and healthcare providers to healthcare-associated infections.

## Zusammenfassung

**Hintergrund:**

Die Qualität der Gesundheitsversorgung in Gesundheitseinrichtungen hängt von der Umsetzung und Einhaltung eines wirksamen Programms zur Infektionskontrolle durch die Betreiber ab. Unser Ziel war es, den Grad der Umsetzung von Richtlinien zur Infektionsprävention und -kontrolle (IPC) in Gesundheitseinrichtungen in einem Land mit niedrigem Einkommen zu bewerten.

**Methode:**

Es wurde eine Querschnittsstudie in 18 Gesundheitseinrichtungen des Gesundheitsbezirks Popokabaka in der Demokratischen Republik Kongo durchgeführt. Die Daten wurden anhand des von der Weltgesundheitsorganisation (WHO) entwickelten IPC-Bewertungsrahmens erhoben und analysiert. Der Beurteilungsrahmen bestand aus acht verschiedenen Abschnitten, von denen jeder mit bis zu 100 Punkten bewertet wurde. Das Niveau der Umsetzung in jeder Einrichtung wurde anhand eines Punktesystems bewertet: unzureichend (0–200), grundlegend (201–400), mittelmäßig (401–600) und fortgeschritten (601–800).

**Ergebnisse:**

Der Medianwert aller Einrichtungen lag bei 181,3 (Interquartilsabstand 145,0–228,1) mit einer Bandbreite von unzureichend bis grundlegend. Zehn (55,6%) Gesundheitseinrichtungen wiesen ein unzureichendes IPC-Umsetzungsniveau auf, während acht (44,4%) ein Basisniveau hatten. IPC-Ausbildung und -Schulung waren die Komponenten, die in den untersuchten Einrichtungen am schlechtesten umgesetzt wurden. Keine dieser Einrichtungen verfügte über multimodale Strategien zur Umsetzung von IPC-Maßnahmen.

**Schlussfolgerung:**

Das Niveau der Umsetzung der IPC-Richtlinien in den Gesundheitseinrichtungen des Popokabaka-Gesundheitsdistrikts bleibt aufgrund der geringen Investitionen in das IPC-Programm niedrig. Das wirkt sich negativ auf die Qualität der Versorgung aus und setzt Patienten und Gesundheitsdienstleister der Gefährdung durch Health-care assoziierte Infektionen aus.

## Introduction

Nosocomial infections are one of the major public-health issues worldwide. They are associated with hospital stay prolongation, antimicrobial resistance, and an increase in healthcare expenditures [1], [2]. Indeed, the World Health Organization (WHO) estimates that healthcare-associated infections (HIAs) affect one in ten hospitalized patients worldwide and two to three times more in low-income countries, resulting in more than three million deaths annually worldwide [3]. In Africa, where 12.7% of hospitalized patients are affected, these infections cause up to 22% of deaths [4]. In the Democratic Republic of Congo (DRC), prevalence estimates ranging from 15–24% were found in various hospitals, leading to deaths and additional healthcare expenditures [5], [6].

According to the WHO, the quality and safety of care in healthcare facilities (HCF) relies on an effective infection prevention and control (IPC) program aligning with recommendations and strategies [2], ([7], p. 24–6), [8], [9]. thereby reducing HAIs by up to 70% [3], [9]. The development of these IPC programs in HCF remains worrying in sub-Saharan Africa. According to the 2022 WHO report on global infection control, only 46.2% of African countries had national programs for the prevention of HAIs with a designated technical team or a focal person [3].

In the Kwango Province Health Division (PHD), only four – including Popokabaka – out of the fourteen health districts had their providers’ capacity built IPC in 2011. In 2022 and 2023, the National Health Information System (SNIS) reported a neonatal infection rate of 15.3% in these health districts and 12.3% throughout the PHD. Neonatal infections are the second most common cause of hospitalization of newborns in neonatology departments in the province [10]. Yet HCFs in the Kwango provincial division are not equipped with a system for assessing the quality of care and monitoring HAIs, and therefore cannot generate evidence.

In this study, we assessed the level of implementation of the WHO guidelines on IPC in the Popokabaka health district, where an IPC program was implemented. Through this study, we identified weaknesses and strengths regarding IPC resources and practices and suggested strategies to contribute to improving the quality of healthcare services.

## Methods

### Study design, period and site

A descriptive cross-sectional study was conducted in July 2024 in HCFs of the Popokabaka health district. The district is located in the southwestern part of the DRC and is one of the fourteen health districts of the Kwango province health division. It covers more than 200,000 inhabitants and includes a general reference hospital, seven reference health centers, and twenty-four health centers. 

### Sampling

The HCFs (Health Centers, Reference Health Centers and Reference General Hospital) listed in the National Health Information System were included in this study. The service availability and readiness assessment approach was employed to determine the sample size using the formula [11]:


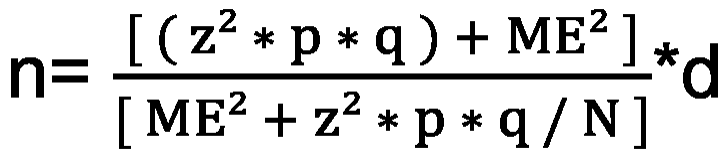
, where:

n = sample size

z = 95% confidence level (1.96)

ME = margin of error 15%

p = the expected proportion of HCFs having an IPC program with well-defined objectives; this study used a proportion of 33.9%, as reported in a study in Ghana [12]. 

q = 1–p (1–0.339=0.661)

d = design effect = 1.0

N: number of HCFs in the Popokabaka health district (32), including one reference general hospital (RGH), seven reference health centers (RHC) and twenty-four health centers (HC).

The sample size was estimated at 17.8 and rounded to 18 health facilities. To select HCFs for the survey, eligible HCFs were stratified based on their type into health centers, reference health centers, and reference general hospital. In each stratum, HCFs were selected using a probability proportional to their size. Thirteen health centers, four reference health centers, and one reference general hospital were selected. 

### Data collection

Data were collected using a structured, closed-format questionnaire with associated score calculation on Kobocollect. Kobocollect is a tool designed for the basic evaluation of the IPC program and activities in healthcare facilities, and is based on the IPC assessment framework developed by the WHO, allowing data collection on 81 program indicators grouped into eight sections representing the main IPC program components [13]. 

A total of six experienced surveyors with a good knowledge of the health system were selected and trained for two days. Structured interviews were held with program managers, desk reviews of IPC record files and documents were performed, and IPC practices by healthcare providers were observed. 

### Data processing and analysis

Data were extracted from the server as CSV files, cleaned, and exported to SPSS 27 for analysis.

Categorical variables were summarized as frequencies and proportions. To assess the implementation level of guidelines in HCFs, data were analyzed following the IPC Assessment Framework instructions, which assign a score to each indicator. Based on the level reached by the HCFs for a component, scores ranging from 0–100 were expected. Scores of all components were summed to obtain an overall HCF score varying from 0 to 800. Depending on the overall score obtained, HCF levels were classified as follows:

- Inadequate, if the score was 0–200, indicating deficient implementation and the need for significant improvement.
- Basic, if the score was 201–400, indicating that some aspects of the IPC core components are in place but not sufficiently implemented, thus requiring further improvement.
- Intermediate, if the score was 401–600, suggesting that most aspects are well implemented; that the mechanism should continue to improve the scope and quality of implementation; and that the focus should be on developing long-term plans to support further and promote existing program activities.
- Advanced, if the score was 601–800, indicating that the main components are fully implemented per the WHO recommendations and adapted to the facility’s needs.

### Ethical considerations

The Kinshasa School of Public Health Ethics Committee approved the study (ESP/CE/51/2024). The respondents participated in the study voluntarily and freely after receiving all the necessary information and providing written informed consent. Respondents’ data were kept confidential. They faced no significant risk by participating in the study, and none were forced to participate or reprimanded for refusing. Guidelines and other valuable documents on IPC were provided to the surveyed HCFs. 

## Results

### Baseline data

Overall, 18 healthcare facilities were surveyed. Most respondents were nurses (72.2%), had professional experience of more than five years (55%), and were trained in IPC (77.8 %). Of all the surveyed healthcare facilities, 13 (72.2%) were health centers, followed by 4 (22.2%) reference health centers, and only one reference general hospital. Regarding bed capacity, 14 (77.8%) HCFs had =25 beds, while three (16.7%) had 26-50 beds, and only one had a capacity of more than 76 beds (Table 1 (Tab. 1)).

### Infection prevention and control programs

Only one HCF had an IPC program with clearly defined responsibilities and an annual work plan; 15 (83.3%) had an IPC program without a clear definition of responsibilities, and 8 (44.4%) did not have a team with IPC professionals. Most HCFs (17; 94.4%) had no IPC program budget (Table 2 (Tab. 2)). 

### Infection prevention and control guidelines

Guidelines on standard precautions, hand hygiene, waste management, and prevention of surgical site infections are primarily available in the health district at 14 (77.8%), 17 (94.4%), 7 (38.9%), and 4 (22.2%) healthcare facilities, respectively. None of the HCFs involve stakeholders in the adaptation of these IPC guidelines (Table 3 (Tab. 3)). 

### Infection prevention and control education and training

Most HCFs (17, 94.4%) organized training on IPC irregularly, while only one periodically evaluated the effectiveness of training programs, and no HCF had a continuing education program on IPC. Two HCFs (11.1%) integrated IPC training into clinical practice, whereas no HCFs organized specific training for patients or family members (Table 4 (Tab. 4)).

### Healthcare-associated infection surveillance

It was found that 14 (77.8%) HCFs included surveillance in their IPC program and that in 13 (72.2%) HCFs, professionals in charge of surveillance activities had training in basic epidemiology, surveillance, and IPC. Surgical site infections were monitored in 16 (88.9%) HCFs, and infections were clinically defined in 16 (88.9%) as well. None of the surveyed HCFs monitored infections associated with medical devices and infections among professionals in care units, laboratories, or other services. None of the HCFs had a functional microbiology laboratory to support surveillance (Table 5 (Tab. 5)). 

### Monitoring/audit of IPC practices and feedback and control activities

None of the surveyed HCFs uses multimodal strategies to implement IPC interventions.

Only one HCF has a monitoring plan with clear objectives, targets, and activities, and 15 (83.3%) monitor antimicrobial drugs, or soap for hand hygiene. Compliance with hand hygiene, disinfection, and sterilization of medical devices was poorly practiced in 3 (16.7%) and 1 (5.6%) HCFs. 

None of the HCFs conduct a self-assessment of hand hygiene using the WHO model (Table 6 (Tab. 6)).

### Workload, staffing and bed occupancy at the facility level

In 13 (72.2%) HCFs, there were no assessments for staffing professionals based on the workload. One-third of surveyed HCFs (33.3%) maintained the healthcare professional: patient ratio in more than 50% of units. Eleven (61.1%) HCFs complied with the standard of one patient per bed, while 15 (83.3%) HCFs claimed to respect bed spacing of one meter (Table 7 (Tab. 7)).

### Built environment, materials and equipment for IPC at the facility level

Eleven (61.1%) HCFs claimed to have water on average ≥5 days per week or every day, but not in sufficient quantity, whereas 18 (88.9%) had hand hygiene stations but without permanently available consumables. Of all the surveyed HCFs, none had functional toilets/latrines of sufficient quality. Moreover, none of the HCFs had traceability of floor and surface maintenance, and only 15 (83.3%) had appropriate equipment for cleaning, but they were not well maintained (Table 8 (Tab. 8)). Fourteen (77.8%) HCFs had waste collection containers but were not up to standard, and 6 (33.3%) had a functional landfill/fenced waste dump or municipal collection. Ten (55.6%) HCFs did not have an incinerator or alternative technology for treating infectious and perforating/sharp waste. Most HCFs (17, 94.4%) did not have a disinfection sector and/or a central medical-device sterilization unit (Table 8 (Tab. 8)). 

Overall, 10 (55.6%) HCFs had an inadequate level of IPC, including 9 (69.2%) health centers and 1 (25.0%) reference health center. On the other hand, eight (44.4%) HCFs had a basic level of infection prevention and control, including three (75%) reference health centers, the general referral hospital, and four (30.8%) health centers. The highest-rated components in the surveyed healthcare facilities were: workload, staffing, and bed occupancy 45.0 (38.8–66.3), surveillance of HAIs 35.0 (29.4–42.5), built environment, materials and equipment for IPC at the facility level 27.5 (17.5–32.5). IPC guidelines 8.8 (5.0–17.5), education and training were poorly implemented. None of the surveyed HCFs had used multimodal strategies to implement IPC interventions (Table 9 (Tab. 9)).

## Discussion

The provision of quality healthcare services in facilities depends on the establishment of effective IPC programs that comply with WHO guidelines ([7], p. 24–6), [14], [15]. We evaluated the level of implementation of WHO guidelines on IPC in healthcare facilities in the Popokabaka health district, using the WHO’s IPC Assessment Framework. 

The study revealed that healthcare facilities in the Popokabaka health district have an IPC level that does not exceed “basic”. The low level of implementation of these guidelines, as observed, may result from the absence of stakeholder ownership strategies, the lack of prioritization of IPC activities within the healthcare system, insufficient investments and support, and underdeveloped health policies in the field of IPC.

It appears that the facilities in the Popokabaka health district face similar challenges to those of low-income countries, as listed by WHO [3]. Although this study was conducted only in peripheral-level facilities, the results are similar to those found in Ghana [12] and in facility level in underdeveloped areas of Pakistan [16], where the majority of facilities had inadequate or basic levels.

In general, health centers showed a lower level of implementation compared to reference health centers and the general reference hospital. The disparities observed across different categories of facilities could be due to inequalities in resource allocation between these structures; although they are all peripheral-level facilities, reference facilities are prioritized in certain interventions. This situation could also be explained by the low structural development of health centers in the health district. These results are similar to those obtained in Niamey, Niger [15], where health centers had low performance levels, but our results differ from those obtained in the study conducted in Kinshasa, DRC [17], where health centers overall had a performance level of 71% (intermediate). This performance is probably linked to the significant support received by healthcare facilities in highly affected provinces during the COVID-19 pandemic, including Kinshasa.

Regarding the organization of the IPC program, the absence of IPC programs with well-defined objectives in the majority of facilities results from a glaring lack of IPC professionals in the health district who can ensure the development of this program while investing in human resources. The lack of budget allocation for IPC activities in the majority of facilities is a consequence of the non-prioritization of these activities in facilities, which generally operate with limited means derived from local production due to a lack of subsidies or operating funds. These results are similar to those obtained in Ghana [12], where only 33.9% of facilities had an IPC program with clearly defined objectives.

Assigning IPC professionals capable of fully dedicating themselves to these activities is necessary; although financial and material investments in IPC are required, it is also essential to raise awareness among facility managers about the importance of prioritizing IPC activities.

The lack of guidelines on key infection control measures could be explained by limited access to information in the area, a shortage of IPC professionals capable of adapting these guidelines to local realities, and a lack of dissemination of these guidelines by the intermediate level. These results are consistent with those found by the WHO, stating that, on average, only 32.3% of facilities in Africa had key infection-control guidelines, whereas in Ghana [12], the majority of facilities had these guidelines. This discrepancy can be explained by the higher level of access to information and new technology in Ghana compared to the DRC.

The lack of education and continuous training programs in healthcare facilities is one of the reasons behind the low performance observed in infection control in these facilities. This situation partly results from a traditional educational system, in which IPC programs are not integrated into the curriculum at various levels, as well as from the limited investment by facilities in the continuous training of providers. In a study conducted in Kinshasa, it was found that continuous IPC training for providers and education for visitors were not conducted in any facility, whereas in Ghana [12] and Niger [15], respectively, 35.7% and 71% of facilities organized periodic training on IPC guidelines for staff.

The underdevelopment of healthcare-associated infection surveillance in facilities, coupled with the limitation of surveillance to a single indicator (surgical site infections), results from the non-integration of HAIs surveillance into the national health information system, where efforts and investments are focused only on epidemiological surveillance. The absence of a microbiology laboratory in the health district stems from the national health policy, where only provincial reference facilities are prioritized for establishing laboratories capable of performing microbiological cultures. This situation means that infections are only clinically defined in facilities. Our results are consistent with those of the WHO [18], which found that only 18% of low-income countries globally had a plan and monitored HAIs surveillance indicators, and none of these countries had a reference laboratory at the primary level to support surveillance. However, the high rating of staff responsible for infection surveillance in the surveyed facilities likely results from capacity-building efforts conducted within the framework of epidemiological surveillance. Ideally, IPC activities should be integrated into the national health information system (NHIS) and receive sustained attention to improve the quality of care provided.

### Follow-up and feedback of IPC practice audits

The absence of a self-assessment culture and follow-up plans with clear objectives, targets, and activities in facilities prevents them from improving over time. The lack of expertise in this area and the lack of involvement of higher levels could explain this situation, which requires the involvement of managers and administrators.

### Built environment, materials, and equipment for IPC in the surveyed facilities

The deficiencies observed in water, hygiene, and sanitation in the facilities reflect the low level of development of infrastructure dedicated to IPC in the health district. As most facilities do not have a disinfection sector and/or a central sterilization service for medical devices, patients are at a high risk of HAIs in these facilities. These results are similar to those found in a study conducted in Niger [15], where hand hygiene facilities were nonexistent. However, these results do not align with those found in the WHO's global survey [18], where 68% of healthcare facilities in low-income countries had hand hygiene facilities and daily and sufficient water services.

The difference to the WHO’s survey may result from the settings where the studies were conducted, as our study was only conducted in rural facilities. The results of our study can be explained by the lack of clear and significant investment in IPC.

The implementation of the WHO’s IPC guidelines still poses challenges in most of the surveyed facilities. These programs have not been developed, highlighting the importance of significant improvements by allocating sufficient resources for IPC, strengthening the capacities of healthcare providers, and developing the managerial skills of various healthcare facility administrators to integrate IPC into all routine activities.

## Conclusion

Our study revealed that the implementation level of IPC guidelines in healthcare facilities of the Popokabaka health zone remains low. Factors such as the poor investment of resources in IPC programs, the absence of training programs and education of providers on IPC, the lack of guidelines, and the non-use of multimodal strategies significantly contribute to perpetuating this low level of implementation. We recommend mobilizing stakeholders and more resources, as well as establishing programs to strengthen providers’ capacities to improve the quality and safety of service delivery.

## Notes

### Competing interests

The authors declare that they have no competing interests.

### Ethical approval 

The Kinshasa School of Public Health Ethics Committee approved the study (ESP/CE/51/2024). 

### Funding

None. 

### Acknowledgments

We would like to thank the Kinshasa School of Public Health faculties, the Kwango provincial health division, healthcare workers, and managers of health facilities in the Popokabaka health district. We are also grateful to Drs. Didier TSIKISA, Jean-Paul MIKORY, Gloria BISIMWA, Grace KABENGELE, Yannick MUNYEKU and Sakarine KIESE for their contribution in carrying out this work.

### Authors’ ORCID

- Fraste Muswiya Kaswij: 0009-0006-0722-1078
- Martin Mutuza Bakuzeza: 0009-0001-3081-3526
- Dalau Nkamba Mukadi: 0000-0002-5379-0507

## Figures and Tables

**Table 1:**
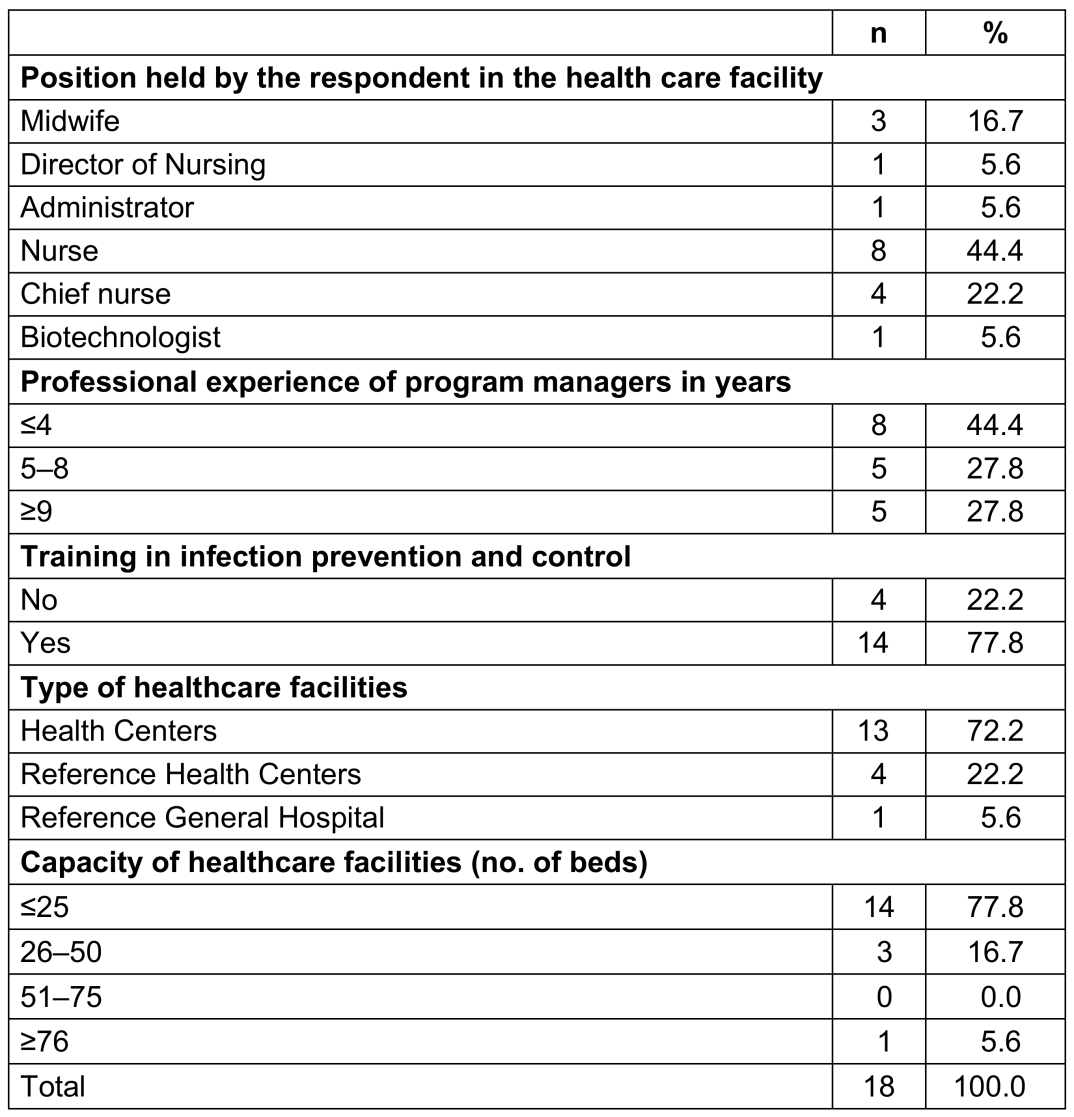
Profile of providers and organizational characteristics of healthcare facilities

**Table 2:**
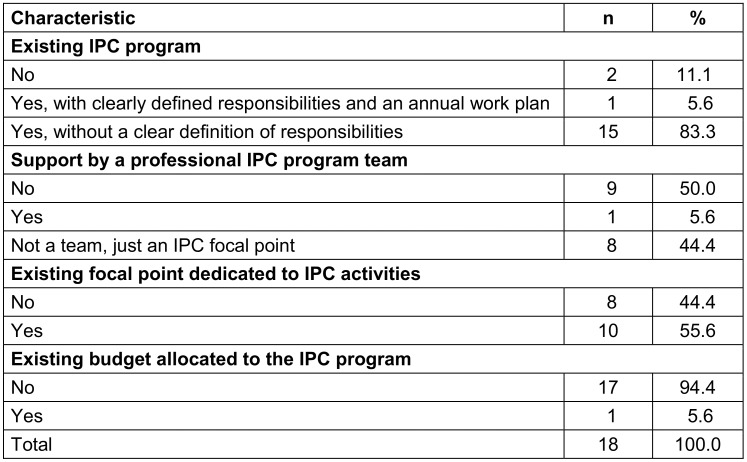
Management of infection prevention and control program in healthcare facilities

**Table 3:**
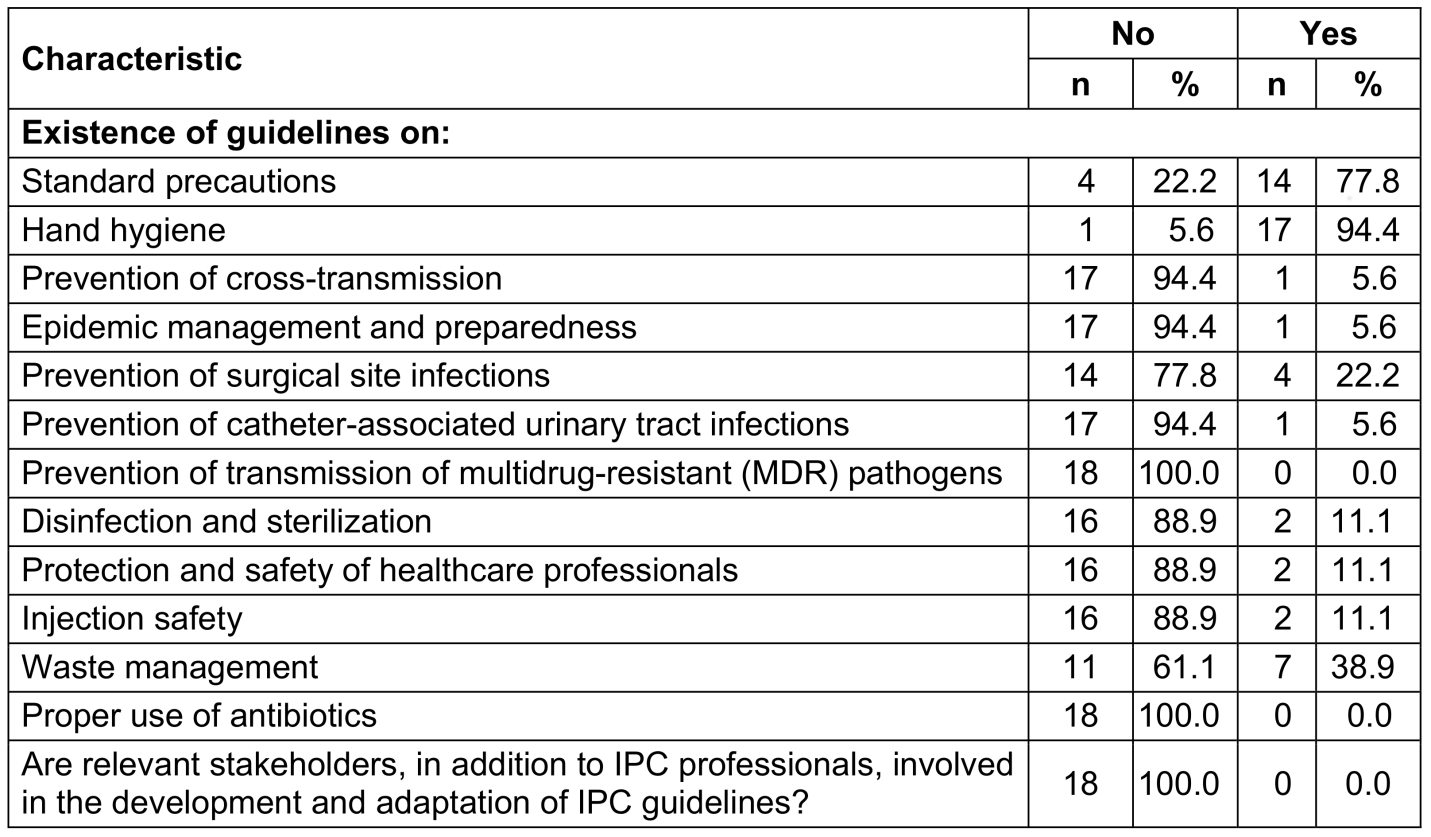
Availability of guidelines in healthcare facilities

**Table 4:**
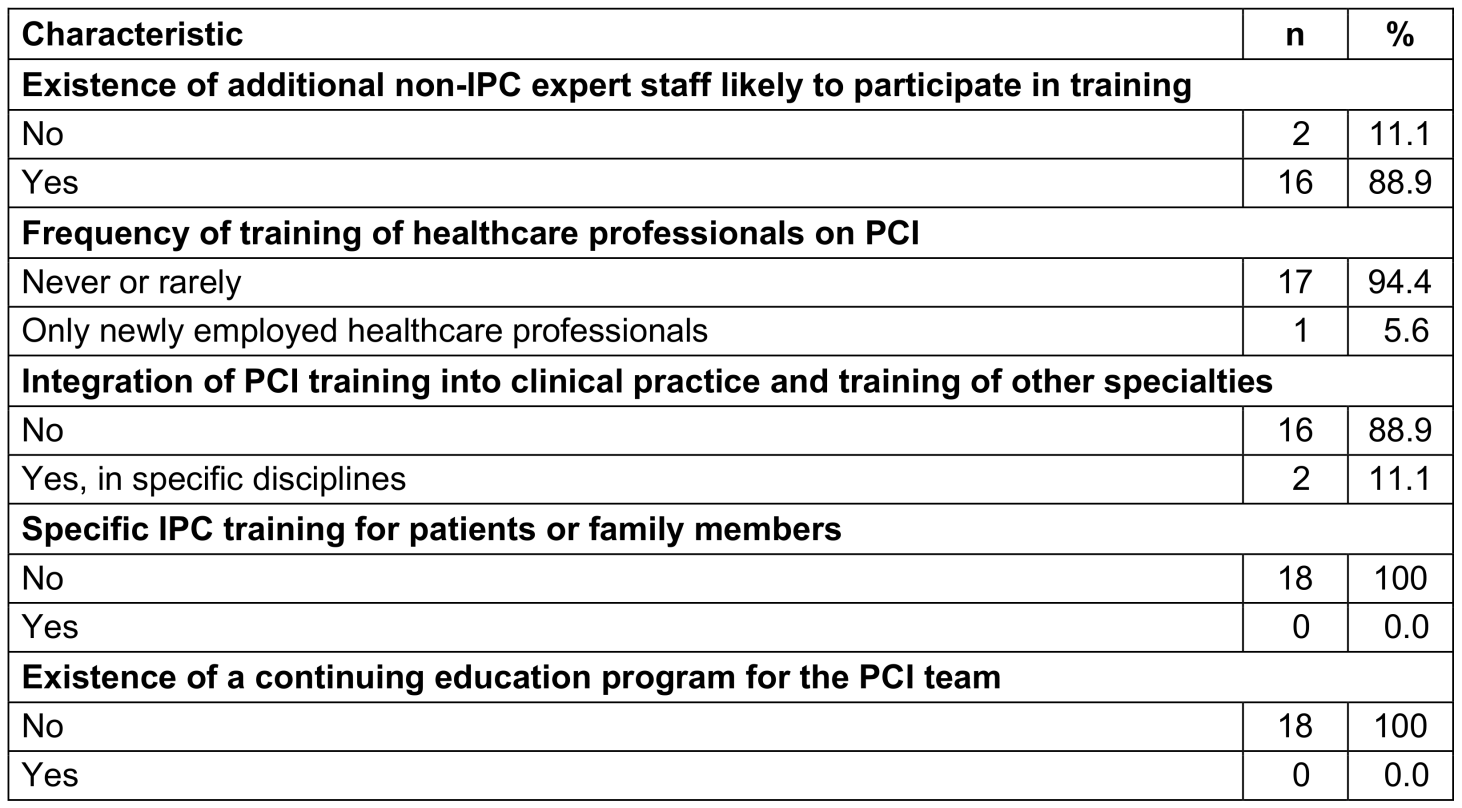
Organization of education and training on infection prevention and control in healthcare facilities

**Table 5:**
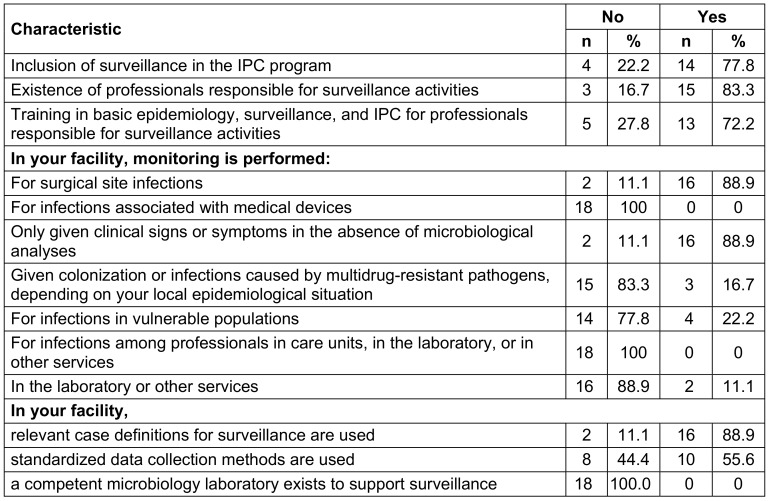
Surveillance of healthcare-associated infections

**Table 6:**
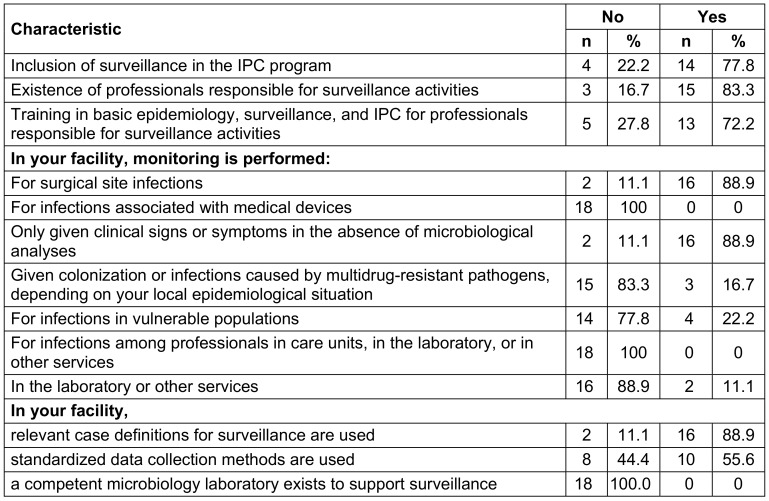
Organization of multimodal strategies, monitoring and audit reporting of infection prevention and control practices in healthcare facilities

**Table 7:**
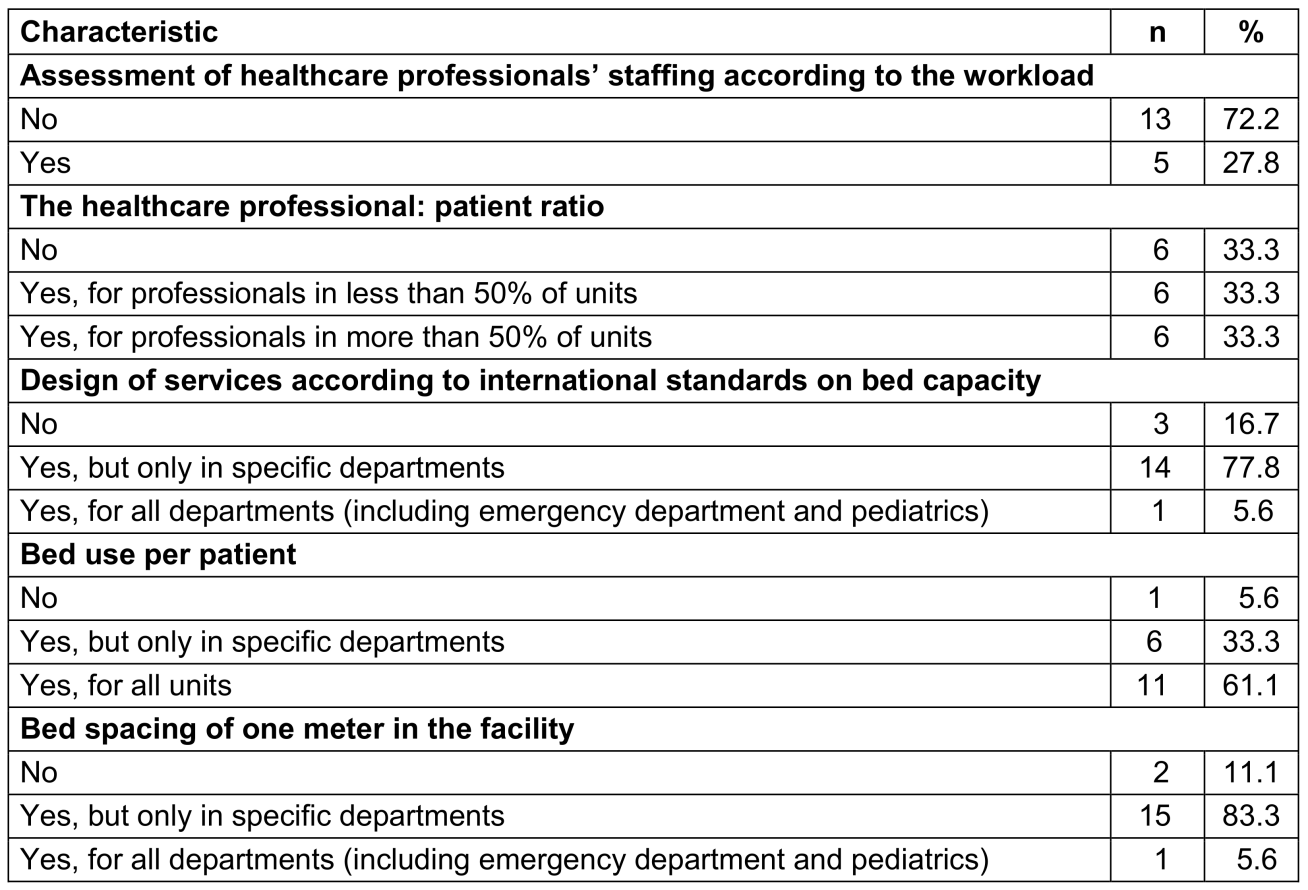
Organization of workload, staffing, and bed occupancy in healthcare facilities

**Table 8:**
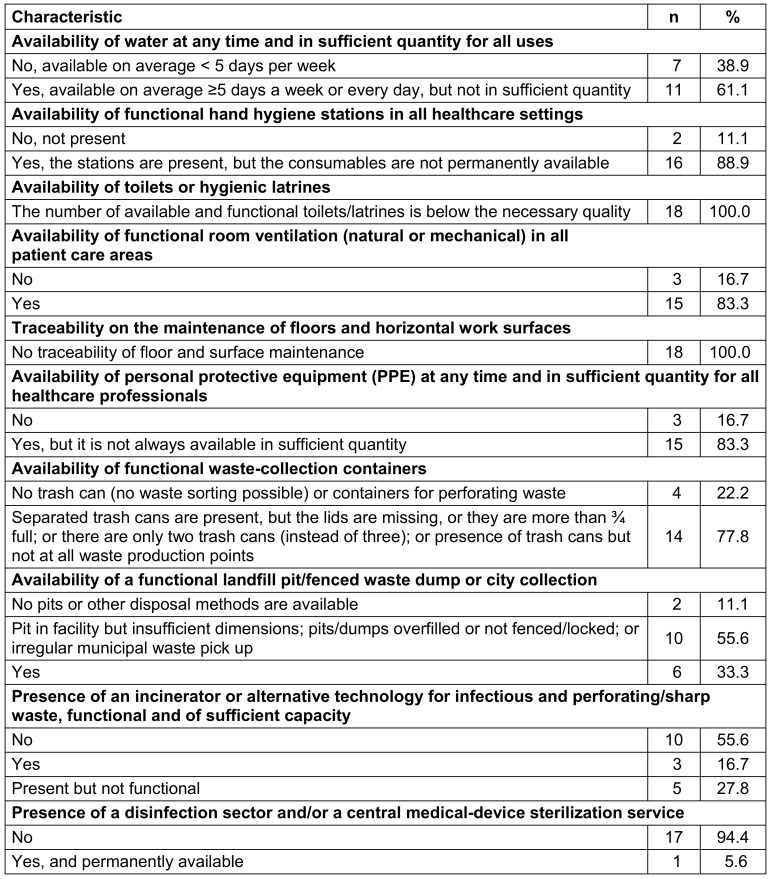
Description of the built environment, materials, and equipment for preventing and controlling infection in healthcare facilities

**Table 9:**
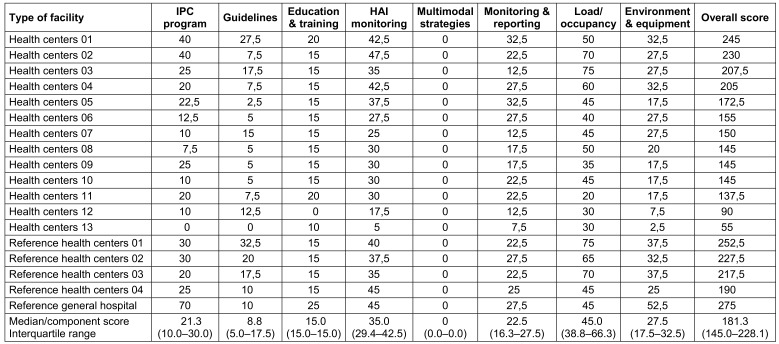
Distribution of score by main components of infection control in the surveyed healthcare facilities
